# Unidimensional measurement may be superior to assess primary tumor response after neoadjuvant chemotherapy for nasopharyngeal carcinoma

**DOI:** 10.18632/oncotarget.14941

**Published:** 2017-02-01

**Authors:** Chuanben Chen, Xiurong Lin, Yuanji Xu, Penggang Bai, Youping Xiao, Yuhui Pan, Chao Li, Zhizhong Lin, Mingwei Zhang, Yunbin Chen

**Affiliations:** ^1^ Department of Radiation Oncology, Fujian Cancer Hospital, Fujian Medical University Cancer Hospital, Fuzhou, Fujian, China; ^2^ Shengli Clinical Medical College of Fujian Medical University, Fuzhou, Fujian, China; ^3^ Fujian Provincial Key Laboratory of Translational Cancer Medicine, Fuzhou, Fujian, China; ^4^ Department of Radiology, Fujian Cancer Hospital, Fujian Medical University Cancer Hospital, Fuzhou, Fujian, China; ^5^ Department of Radiotherapy, First Affiliated Hospital of Fujian Medical University, Fuzhou, Fujian, China

**Keywords:** nasopharyngeal carcinoma, neoadjuvant chemotherapy, tumor measurement, magnetic resonance imaging

## Abstract

Application of current response evaluation criteria in solid tumors (RECIST 1.1) for assessment of irregularly shaped nasopharyngeal carcinoma (NPC) is a gray area with much ambiguity. Our aim was to compare unidimensional measurements (UDM) and bidimensional measurements (BDM) on magnetic resonance images in alternative planes for measurement of tumor response after neoadjuvant chemotherapy (NACT) in patients with locally advanced NPC. 59 patients with untreated non-metastatic NPC were prospectively enrolled. The size or change in size of the primary tumor and retropharyngeal nodes was assessed by UDM and BDM on axial and coronal planes before and after 2 cycles of NACT. Tumor volume was considered as the reference standard. Correlation between volume and diameter was analyzed using a general linear model. The degree of agreement and discordance of response classification based on different measures were evaluated with κ statistic and McNemar's test, respectively. Both axial UDM (RECIST 1.1) and axial BDM (WHO) showed a significant association with volumetric standard. However, the agreement of axial UDM with VM was better than that of axial BDM (κ value: 0.514 to 0.372). In addition, when increasing coronal planes to evaluate tumor response with UDM and BDM, an inferior agreement between coronal BDM and VM was still observed. Notably, coronal UDM showed the best consistency with volume (κ = 0.585). Hence, axial UDM showed better correlation with volumetric measurements than axial BDM. Since coronal UDM showed high correlation to VM, we suggest further research to assess its use for response assessment of NPC after NACT.

## INTRODUCTION

Nasopharyngeal carcinoma (NPC) represents a particular therioma with irregular infiltration into the surrounding soft tissues, which causes difficulty for clinicians to evaluate therapeutic response. Neoadjuvant chemotherapy (NACT) has repeatedly been shown to reduce the risk of recurrence and distant metastasis in patients with locally advanced NPC [1–3]. Therefore, it is important to precisely evaluate the efficacy of NACT. Currently, assessment of tumor size and change in size in clinical trials is performed based on the World Health Organization (WHO) criteria and the Response Evaluation Criteria in Solid Tumors (RECIST), which is based on bidimensional measurements (BDM) and unidimensional measurements (UDM) on axial planes, respectively [4, 5]. To our knowledge, the only comparative study of different measures based on response evaluation criteria in NPC was conducted by King et al., who found that the BDM to be superior to UDM for evaluation of therapeutic response in patients with irregularly shaped nasopharyngeal tumors [6]. In recent studies, however, UDM was constantly employed to evaluate the efficacy of NACT for NPC, and tumor response to NACT was shown to be closely associated with the prognosis [7, 8]. This has created some confusion over the choice of methods for evaluation of therapeutic efficacy in these patients. Therefore, verification of the practicability of these two techniques is a key imperative.

In 1981, the WHO criteria were first published to estimate tumor size and response by summing the products of bidimensional lesion measurements on axial images [4]. However, some of the parameters and criteria in the WHO standard such as the minimum size of the lesion, the number of lesions recorded, and criteria for disease progression were not well defined. In 2000, the RECIST 1.0 criteria were developed to adopt unidimensional measurement on axial planes to facilitate and refine the tumor response measurement. The RECIST criteria were updated in 2010, with a more detailed definition of measurable and non measurable lesions and disease progression, and to discuss the optimal anatomical assessment of lesions [5]. RECIST guidelines have become more and more popular as its standards are continuously improved to meet the needs of the research. However, their use for evaluation of irregular tumors including malignant pleural mesothelioma and recurrent malignant glioma was not successful [9, 10]. Hence, whether the new RECIST criteria can be applied to assess irregularly shaped tumors remains largely unknown, especially in the era of volumetric measurement.

Although the BDM was shown to be superior to the UDM for assessment of tumor response in patients with NPC [6], some deficiencies existed in this study. First, the number of NPC patients who received NACT was only 17, which limited the statistical power of the analysis. In addition, assessment of retropharyngeal lymph nodes (RLN) was not performed in that study. Some RLNs are difficult to identify as they are often merged with the primary tumor. A close correlation between RLN metastasis and parapharyngeal space involvement as well as metastasis to lower neck nodal levels was reported [11]. Therefore, the approach used by King et al. needs to be modified to include both the measurements of primary tumor and RLN as described in other studies [12, 13]. Furthermore, the techniques are no longer confined to the axial images with increased use of magnetic resonance image (MRI). The alternative plane to use BMD or UDM may also be sagittal or coronal. Therefore, further research is needed to determine which alternative plane may be used to measure primary tumor and RLN in NPC.

In the present study, we use MRI to investigate if the BDM or UDM on the axial plane would authentically reflect tumor size and alteration in size after 2 cycles of NACT in a large cohort of patients with locally advanced NPC. New measurements such as BDM or UDM on coronal images were assessed with regard to tumor size and change in size with NACT as well. VM served as the reference standard, which was automatically obtained from the 3D image-based treatment planning system.

## RESULTS

### Patient characteristics

The study group consisted of 59 (39 men and 20 women; median age: 46 y [range, 18-65]) with locally advanced NPC: T1 (n = 9); T2 (n = 10); T3 (n = 26); T4 (n = 14). Of these, 56 patients had developed retropharyngeal lymph node metastases. The male to female ratio was 1.95:1. Patient characteristics including histopathology, clinical stage, and chemotherapy regimens are summarized in Table 1.

**Table 1:** Characteristics of patients with locally advanced NPC

Parameters	Number of patients	%
Gender		
Male	39	66.1
Female	20	33.9
Age, years^a^		
<46	28	47.5
≥46	31	52.5
Histopathology^b^		
Type II	4	6.8
Type III	55	93.2
T classification		
T1	9	15.3
T2	10	16.9
T3	26	44.1
T4	14	23.7
RLN involvement		
Yes	56	94.9
No	3	5.1
AJCC stage		
III	41	69.5
IVa-IVb	18	30.5
NACT regimen		
PTX + nadeplatin	42	71.2
GCB + nadeplatin	17	28.8

Note: ^a^Median 46; range 18-65 years.

^b^According to the World Health Organization type.

Abbreviations: RLN: retropharyngeal lymph nodes; AJCC: American Joint Committee on Cancer; NACT: neoadjuvant chemotherapy; PTX: paclitaxel; GCB: gemcitabine.

### Tumor size at diagnosis

Tumor volumes at first diagnosis were automatically obtained from the 3D treatment planning system (range: 6.0 to 206.2 cm^3^; median: 22.7 cm^3^; interquartile range: 24.1 cm^3^). The inter-observer reliability for VM, Ax-UDM, Cox-UDM, Ax-BDM, and Cor-BDM is shown in Table 2. The ICCs for VM were significantly higher, and that for Ax-UDM were slightly higher, as compared to Cor-UDM, Ax-BDM, and Cor-BDM, respectively. The correlation of Ax-UDM, Cox-UDM, Ax-BDM, and Cor-BDM with VM is shown in Table 3. All 4 measures showed a significant association with VM at initial diagnosis, according to their probability values.

**Table 2:** Intraclass correlation coefficients for different measurements

	ICC (95%CI)	*P*-value
VM^pre^	0.997 (0.996-0.998)	<0.001
Ax-UDM^pre^	0.969 (0.948-0.981)	<0.001
Cor-UDM^pre^	0.921 (0.868-0.953)	<0.001
Ax-BDM^pre^	0.948 (0.913-0.969)	<0.001
Cor-BDM^pre^	0.945 (0.908-0.968)	<0.001

Abbreviations: ICC: intraclass correlation coefficient; VM: volumetric measurement; Ax-UDM: unidimensional measurements in axial planes; Cor-UDM: unidimensional measurements in coronal planes; Ax-BDM: bidimensional measurement in axial planes; Cor-BDM: bidimensional measurement in coronal planes.

**Table 3:** Probability values for associations of different diameter measurements with VM

	VM at diagnosis	VM after NACT	Percentage change in VM after NACT
Ax-UDM	<0.001	<0.001	<0.001
Cor-UDM	<0.001	<0.001	<0.001
Ax-BDM	<0.001	<0.001	<0.001
Cor-BDM	<0.001	<0.001	<0.001

Abbreviations: VM: volumetric measurement; NACT: neoadjuvant chemotherapy; Ax-UDM: unidimensional measurements in axia planes; Cor-UDM: unidimensional measurements in corona planes; Ax-BDM: bidimensional measurement in axia planes; Cor-BDM: bidimensional measurement in coronal planes.

### Evaluation of tumor response

As for tumor response, for one thing, with regard to absolute change in size and percentage change in size after treatment with NACT, Ax-UDM, Cor-UDM, Ax-BDM, and Cor-BDM were still found to be significantly associated with VM (Table 3).

For another, tumor response according to all 4 measures and VM was also evaluated by the κ analysis and McNemar's test (Table 4). As for tumor response based on the VM standard, there were 10 PR and 49 SD with no CR and PD. As for tumor response based on Ax-UDM (RECIST 1.1), there were 16 PR and 43 SD, and no CR and PD, (κ value: 0.514). To compare with the VM standard, Ax-UDM was shown to misclassify 2 PR cases as SD and misclassify 8 SD cases as PR (*P*< 0.001, McNemar's test). As for tumor response based on Cor-UDM, there were 14 PR and 45 SD (κ value: 0.585). To compare with the VM standard, Cor-UDM was found to misclassify 2 PR cases as SD and misclassify 6 SD cases as PR (*P*< 0.001, McNemar's test). As for tumor response based on Ax-BDM (WHO criteria), there were 21 PR and 38 SD (κ value: 0.372). When compared with the VM standard, Ax-BDM was found to misclassify 2 PR cases as SD and misclassify 13 SD cases as PR (*P*= 0.001, McNemar's test). As for tumor response based on Cor-BDM, there were 24 PR and 35 SD (κ value: 0.381). When compared with the VM standard, Ax-BDM was found to misclassify 1 PR cases as SD and misclassify 15 SD cases as PR (*P*< 0.001, McNemar's test). Therefore, it was not difficult to find that the kappa values of the UDM, either on axial or coronal planes, were markedly higher than that of the BDM on axial or coronal planes, and of which, Cor-UDM seemed to be the highest. When VM was taken as the reference standard, Ax-UDM, Cor-UDM, Ax-BDM and Cor-BDM were finally found to misclassify the tumor response in 10 of 59 cases (16%), 8 of 59 cases (14%), 15 of 59 cases (25%), and 16 of 59 cases (27%), respectively. It was obvious that the BDM measured in alternative planes after NACT accounted for a higher error rate as compared to that with UDM on alternative planes.

**Table 4:** Summary of the kappa values for different measurements

	Kappa value	Misclassify SD as PR	Misclassify PR as SD
Ax-UDM	0.514	8	2
Cor-UDM	0.585	6	2
Ax-BDM	0.372	13	2
Cor-BDM	0.381	15	1

Abbreviations: SD: stable disease; PR: partial response; Ax-UDM: unidimensional measurements in axial planes, Cor- UDM: unidimensional measurements in coronal planes; Ax-BDM: bidimensional measurement in axial planes; Cor-BDM: bidimensional measurement in coronal planes.

## DISCUSSION

Volumetric methods by manual delineation of target areas on 3D cross-sectional images has high repeatability and may provide the most accurate measure of tumor size regardless of the shape of the tumor [14, 15]. Nevertheless, this technique demands a lot of time, a high level of expertise and more manpower that makes it impractical for routine clinical use. New semi-automated measurement technology was used in an effort to make up for these deficiencies, but the results have remained unsatisfactory because of the relatively intensive labor [16, 17]. Therefore, tumor size was usually assessed by simple diameter measurements including bidimensional measurement (WHO criteria) and unidimensional measurement (RECIST criteria). A previous study indicated that the RECIST criteria (version 1.0) may not be applicable to irregularly shaped nasopharyngeal cancers [6]. However, this trend may be altered with improvements in the new RECIST criteria (version 1.1). Furthermore, taking an alternative plane for UDM or BDM is likely to become more feasible with widespread use of 3D MR imaging. In the present study, both Axial UDM (RECIST 1.1) and axial BDM (WHO) showed strong correlation with VM at diagnosis, absolute change, and percentage change on assessment of primary tumor and retropharyngeal nodes in patients with NPC. However, the agreement of tumor response between axial UDM and VM was better than that between axial BDM and VM. In addition, it is noteworthy that the new measurement using coronal UDM was the most consistent with the volumes.

For comparison of tumor size measurements at diagnosis, VM showed the highest ICCs between the observers compared with the other 4 diameter measurements, in spite of their significantly high ICCs. This result revalidated the high reproducibility of VM which could certainly be taken as a reference standard for UMD or BDM of tumor size. In addition, both UDM and BDM showed a significant association with VM at diagnosis, on both axial and coronal MR imaging planes (all *P*< 0.001). Primary tumor volume was shown to be an independent prognostic factor for local control of NPC, which may appear to be more predictive than T classification [18–20]. However, in recent years, simple diameter measurements were also be used to evaluate the primary tumor volume at diagnosis. Chang *et al*. found that BDM of primary tumor and retropharyngeal nodes for NPC was also significantly correlated with VM at first diagnosis on MRI, and that BDM was shown to independently predict distant metastasis and overall survival [13]. Liang *et al*. reported that UDM for NPC was an independent prognostic factor for all endpoints of disease survival including local control, distant metastasis, and overall survival [21]. Collectively, the simple measurements including UDM or BDM may be further considered for incorporation into the current staging system for NPC to improve the prognostic significance.

With respect to tumor response, all 4 diameter measurements for absolute change in size and percentage change in size after NACT were still significantly associated with VM (all *P*< 0.001). However, with respect to tumor response assessed by the κ analysis and McNemar's test, it was observed that Ax-UDM had a better κ value than Ax-BDM (0.514 vs. 0.372), whereas it had lower misclassification rate compared with Ax-BDM (16% vs. 25%). These findings suggest that the current RECIST 1.1 criteria may be superior to WHO criteria for assessment of tumor response to NACT for irregularly shaped NPC. Our results are contrary to those reported by King *et al*., who found Ax-BDM to be more reliable to reflect tumor response to treatment despite adopting Ax-UDM according to the RECIST 1.0 [6]. All the same, the corresponding explanations have not been well documented in that study. Indeed, the RECIST criteria are based on the theory that the sum of the longest diameters was more coincident with the logarithm of cell number than the sum of bidimensional products [22]. This theory was supported in the context of different types of cancers including both spherical and nonspherical shaped carcinomas such as breast cancer, melanoma, soft tissue sarcoma, and colorectal cancer [22, 23]. Therefore, it may be reasonable to use the RECIST 1.1 criteria to evaluate tumor response to NACT for NPC.

It is interesting to note that Cor-UDM possessed the highest κ value of 0.585, which also illustrated a good agreement with VM. However, Cox-BDM showed inferior concordance to volume with associated κ value of only 0.381. This result was also in line with the previous theory that the longest diameter was a closer indicator of the expression of cell death than the products of bidimensional diameters [22]. To our knowledge, NPC is often characterized by extensive infiltration into the adjacent normal tissues, and the lateral and upward invasion are believed to be the most common invasion patterns for locally advanced tumors [24]. The additional coronal largest diameter was usually aligned with the upward invasion, which might result in a good agreement of tumor response according to VM criteria as well as axial largest diameter along with lateral invasion. Furthermore, coronal images have also been shown to be of additional diagnostic value for primary tumor in rectal cancer [25]. These findings suggest that a combination of axial and coronal images may be better to assess tumor response after NACT for NPC in routine clinical practice, which should be validated in a large-scale research.

A concern in clinical practice is whether the bony tumor invasion should be included in the assessment of tumor response when the skull base is involved by NPC. Due to the mostly persistent abnormalities in the bone marrow on MR images after NACT, King *et al*. excluded any skull base invasion from the pretreatment and post-treatment measurements in case of underestimation of tumor response [6]. In fact, the latest RECIST 1.1 guidelines have definitely ruled that bone lesions with identifiable soft tissue components on MRI axial planes can be regarded as measurable lesions [5]. Hence, bony tumor invasion was also excluded from the present study unless it met the definition of measurable bone lesions. Another concern is whether RLNs should be included in the assessment of primary tumor because of their frequent embedment in the primary tumor. In the present study, RLNs were ultimately taken into consideration in the assessment of primary tumor size. One reason was that conglomerated RLNs with primary tumor are difficult to be measured with good objectivity and repeatability even if RLNs were commonly considered as the first order nodes that drain the nasopharynx. Furthermore, RLN metastasis was reported to show a good correlation with parapharyngeal space involvement and cervical lymph nodal metastasis [11]. However, whether the regression pattern of RLNs is more linearly related to that of cervical lymph nodes is not known at present. Finally, RLNs were also excluded in the assessment of target lymph nodal response after NACT for NPC in our previous study [26]; it was, therefore, necessary to take RLNs together with primary tumor to evaluate tumor response.

One limitation of the current study is the lack of resected specimens to be accurately measured because radiotherapy serves as the primary therapeutic strategy for NPC rather than surgery. Of course, the measurements of the volumes of the resected specimens were considered as the gold-standard for measurement of tumor size instead of volume measurements on cross-sectional MR images. Nevertheless, volume measurements showed the highest reproducibility when compared with the other four diameter measurements on MRI, which demonstrated that the volume measurements could be taken as a reference standard in the present study. Furthermore, the increasing use of sagittal plane on MRI has proved to be important in the diagnosis and target delineation of NPC [27]; however, the effect of sagittal measurement in the assessment of tumor response has not been investigated. Due to the limitation of the number of slices, the sagittal measurements were excluded from this study. However, it merits further investigation in a future study.

In conclusion, it is appropriate to use Ax-UDM to assess the load or alteration in the size of the primary tumor and retropharyngeal lymph nodes after NACT for NPC. However, Ax-BDM seems not to be applicable in this respect. In addition, Cor-UDM based on MRI is another feasible measurement; however, the importance of Cor-UDM in the evaluation of tumor response warrants further investigation.

## MATERIALS AND METHODS

### Patient selection

This prospective study was approved by the Institutional Ethics Review Boards at the Fujian Provincial Cancer Hospital (Ref. no. 2015-010-02); written informed consent was obtained from all patients. All patients were enrolled at our hospital. The included criteria were: (1) consecutive patients with pathologically proven, and previously untreated non-metastatic NPC stage III-IVb on the basis of the 2010 AJCC Staging System for NPC [28, 29]; (2) all patients had a Karnofsky score of ≥ 70, and treatment eligible blood counts (white blood cell count ≥ 3600/mm^3^, platelet count ≥ 100,000/mm^3^), hepatic function (serum bilirubin ≤ 1.5 mg/dl) and renal function (serum creatinine of ≤ 1.6 mg/dl); (3) all patients underwent complete MR imaging before and after 2 cycles of neoadjuvant chemotherapy. Patients older than 70, those younger than 18, and those with a prior or synchronous malignancy were excluded. A total of 59 patients met the criteria and were enrolled in the study between September 2014 and February 2016.

### Chemotherapy

All 59 patients received 2 or 3 cycles of neoadjuvant chemotherapy according to their disease stage and physical tolerance. Of which, 44 patients with locally advanced NPC received only 2 cycles of NACT, while 15 patients with N3 stage or with T4 disease involved by extensive invasion of skull base or intracranial tissue were given by 3 cycles of NACT. The specific NACT regimens used were as follows: 42 patients were treated with intravenous paclitaxel (135 mg/m^2^ on day 1) combined with nedaplatin (80 mg/m^2^ on day 2) every 3 weeks; 17 patients received intravenous gemcitabine (1000 mg/m^2^ on day 1 and 8) combined with nedaplatin (80 mg/m^2^ on day 2) every 3 weeks.

### MRI scanning protocol

The MRI scanning protocol used is detailed elsewhere [26]. Briefly, all patients underwent MRI scans in a 3.0-T MultiTransmit Whole Body scanner (Achieva TX, Philips Healthcare, Best, The Netherlands). All MR images were acquired from the central temporal to the thoracic outlet with a 16-channel head and neck combined coil. The routinely used MRI sequences included axial and sagittal fast-spin echo (FSE) T1-weighted imaging (T1WI), axial and oblique coronal FSE T2WI using SPIR technique, and axial and oblique coronal contrast-enhanced (CE) FS T1WI after a bolus injection of 0.1 mmol/kg gadolinium with diethylenetriaminepentacetate (Magnevist, Schering AG, Berlin, Germany). The above MR images of each patient were obtained before and after 2 cycles of NACT.

### Imaging assessment

**Figure 1:**
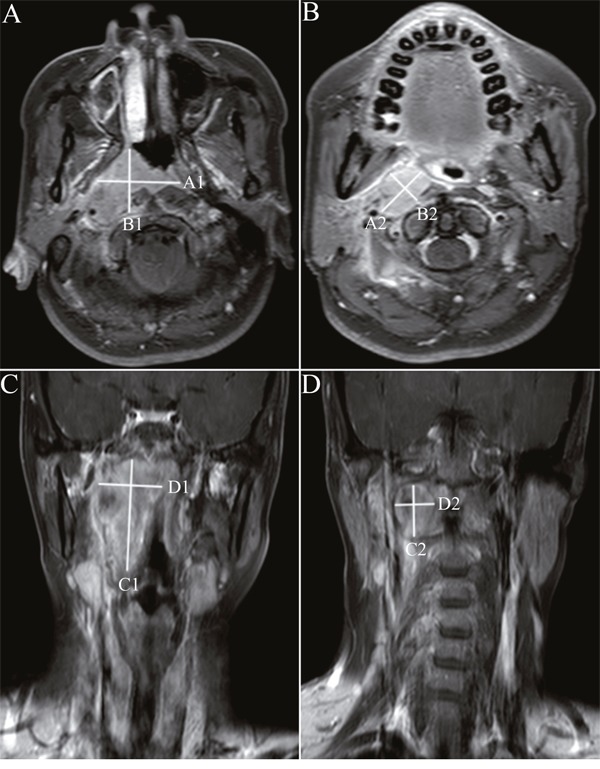
Unidimensional and bidimensional measurements of nasopharyngeal carcinoma in axial and coronal T1-weighted postcontrast MR images Notes: Ax-UDM was obtained by summation of maximum diameter of the primary tumor **A**. and retropharyngeal nodes **B**. in the largest axial slice. Ax-BDM was obtained by summation of the products of the Ax-UDM and the greatest measurement perpendicular to it **(A)** and retropharyngeal nodes **(B)**. Cor-UDM and Cor-BDM of primary tumor **C**. and retropharyngeal nodes **D**. were obtained by the same measurements in the largest coronal slice. Ax-UDM (cm) =A1+A2; Ax-BDM (cm^2^) =A1×B1+A2×B2; Cor-UDM (cm) =C1+C2; Cor-BDM (cm^2^) =C1×D1+C2×D2 Abbreviations: Ax-UDM: unidimensional measurements in axial planes; Ax-BDM: bidimensional measurements in axial planes; Cor- UDM: unidimensional measurements in coronal planes; Cor-BDM: bidimensional measurements in coronal planes.

Measurements of primary tumor and retropharyngeal nodes were independently preformed by two radiologists who specialized in NPC with more than 10 years’ diagnostic experience of MRI. All measurements were conducted with the picture archiving and communication system (PACS). Retropharyngeal nodes with minimal axial diameter of ≥ 5 mm at diagnosis were regarded as malignant lesions which required to be measured. UDM was defined as the measurement of the maximum diameter of primary tumor and retropharyngeal nodes in either axial or coronal planes (Ax-UDM or Cor-UDM); BDM was defined as the product of the UDM and the greatest measurement perpendicular to the UDM in either axial or coronal planes (Ax-BDM or Cor-BDM) (Figure 1). In the event of skull base involvement without identifiable soft tissue components on pretreatment MRI scans, the bony skull base invasion was regarded as non-measurable lesions, as described in the RECIST 1.1 guidelines. In this case, the portion of bony structures was excluded from our measurement range on both pretreatment and post-treatment scans for the purpose of evaluation of tumor response.

### Volumetric measurement

With regard to the VM, the pretreatment and post-treatment MR images of NACT were first transmitted to the 3D treatment-planning system. The area of primary tumor and retropharyngeal nodes were also independently delineated by 2 radiation therapists with more than 10 years of experience in the treatment of NPC. In case of the non-measurable targets because of the involvement of skull base, the corresponding portions were not included. Finally, a 3D image of the delineated lesions was automatically generated by the system and volume was automatically calculated as well.

### Assessment of tumor response

The therapeutic efficacy of NACT was assessed by comparing the changes before and after 2 cycles of NACT. According to WHO, RECIST 1.1, and volumetric criteria [4–6], tumor response is usually categorized into 4 types: complete response (CR), partial response (PR), stable disease (SD) and disease progression (PD). The detailed definitions of the above three criteria are summarized in Table 5.

**Table 5:** Criteria for the assessment of tumor response

Classification	Unidimensional criteria^a^	Bidimensional criteria^b^	Volumetric criteria^c^
CR	Tumor disappearance	Tumor disappearance	Tumor disappearance
PR	>30% decrease in size	>50% decrease in size	>65% decrease in size
SD	Size between that for PR and PD	Size between that for PR and PD	Size between that for PR and PD
PD	>20% increase in size, the sum increase ≥5 mm, the appearance of one or more new lesions	>25% increase in size	>40% increase in size

Note: ^a^Based on RECIST 1.1 guidelines.

^b^Based on WHO guidelines.

^C^According to correlation of alteration in surficial area to alteration in volume.

Abbreviations: CR: complete response; PR: partial response; SD: stable disease; PD: disease progression.

### Statistical analysis

All date analyses were performed using the SPSS version 17.0 statistical software (SPSS Inc., Chicago, IL, USA). A general linear model with univariate analysis was employed to evaluate potential correlation of VM with Ax-UDM, Cox-UDM, Ax-BDM, and Cox-BDM. The correlation coefficient was used to reflect the associations for different diameter measurements with VM. Intra-class correlation coefficient (ICC) was used to assess the test-retest reliability between the different observers for the measurement before and after 2 cycles of NACT. Absolute change and the percentage change were both used to assess tumor response in each group. The tumor response was categorized as CR, PR, SD or PD (Table 5). The degree of agreement of tumor response as assessed by different methods was evaluated by means of κ statistic. McNemar's test was used to compare the error rates of different measurements in the assessment of tumor response. A two-tailed *P* value of < 0.05 was considered statistically significant for all analyses.
